# Changes in haematological and biochemical parameters in blood serum of horses during exposition to workload stress

**DOI:** 10.1016/j.heliyon.2022.e12241

**Published:** 2022-12-13

**Authors:** Martin Massányi, Marko Halo, Peter Massányi, Eva Mlyneková, Agnieszka Greń, Grzegorz Formicki, Marko Halo

**Affiliations:** a AgroBioTech Research Centre, Nitra, Slovak Republic; b Institute of Applied Biology, Slovak University of Agriculepartment of Animal Physiology, Slovak University of Agriculture, Nitra, Slovak Republic; c Institute of Animal Husbandry, Faculty of Agrobiology and Food Resources, Slovak University of Agriculture, Nitra, Slovak Republic

**Keywords:** Horse, Biochemical parameters, Hematology, Stress, Blood

## Abstract

Health state of animals undergoing experimental procedures is an important topic nowadays, as even the small changes can influence the outcome of entire outcomes. Main aim of the study was to evaluate the influence of horse training on variety of blood parameters including mineral profile, energy profile, hepatic profile and haematology. In the experiment, the studied group of horses underwent training programme which consisted of transportation, lounging, riding, jumping, racing, treadmill training and shoeing. Blood samples were collected and later evaluated at the beginning, in the middle and at the end of 1-year lasting process. Our results show multiple significant changes in blood parameters, including changes to multiple minerals, such as Ca, K, Na as well as significant changes of total proteins, urea and certain hepatic profile parameters. Haematology results have also been affected in individual sample collections. Based on results of our study we can state that there have been changes to the internal milieu of the horse but also that there have not been any visible changes of the health status of the animals over the duration of the experiment.

## 1 Introduction

In general, there are multiple types of loads in the daily routine of sports horses that can affect their internal environment. To maintain the quality of the experiment, it is vital to control the animals health in perfect condition to ensure welfare to keep the results from being skewed by inaccuracies that do not correspond with the focus of the experiment [1].

In horse*s, vena jugularis* is the most common location used to obtain blood samples. Obtaining samples from mentioned vein makes it easy to gather samples and therefore also less stressful for experimental animals, which results in minimal external influences to gathered data caused by the sample collection itself. Since there is few information about the health status of animals during experiments following stress observations in horses, it is important to gather data about this subject as horse training for racing purposes is getting more and more sought-after nowadays [2, 3, 4].

Research focused on physiological state of animals in experiments, especially the ones following such sensitive topics as observing stress during training programmes, is the first step to improve the welfare of the animals as well as possible improvements to performance of individual horses [5, 6, 7].

The aim of the study was to observe the health status of animals over a 1-year period, during which the experimental horses were subjected to multiple types of load, targeting to obtain valuable data, which can confirm or deny whether stress caused by day-to-day routine has long lasting effect on internal environment of horse.

## 2 Materials and methods

All experimental procedures and management of animals were conducted in accordance with European Community guidelines m. 86/609/EEC regarding the protection of animals for experimental purpose and experimental conditions were accepted by institutional ethical committee. Blood samples were obtained from horses (n = 10) of sports breeds stabled in Experimental Centre at Institute of Animal Husbandry – 12 males and 2 females and weighed ±550 kg. Experimental animals were stabled in box housing with sawdust litter, fed three times a day with a complete feed mixture [8], that was balanced for individual animals' needs based on their training and weight. Bulk feed was fed in the morning and evening *ad libitum*. The training load set to all the horses one year before the experiment was medium.

### 2.1 Experimental workload types

Over the course of the experiment, animals were regularly and repeatedly subjected to multiple different types of loads:

1. Transport – the group consisted of horses that were transported in pairs for 58 km, duration of the route was 60 min.
2. Treadmill training – horses trained on HorseGym 2000 (HorseGym 2000 GmbH, Harburg-Großsorheim, Germany) training load regulator with exact set training load to: 10 min of step on straight at 6.7 km/h; 10 min of uphill step (3°) at 6.7 km/h; 5 min step uphill (6°) at 6.7 km/h; 15 min stepping on straight at 6.7 km/h.
3. Riding training – tested horses were observed in medium load. Training consisted of basic training work in the duration of 60 min twice a week.
4. Shoeing – Horses were cured by certified farrier for approximately 50 min every 6 weeks.
5. Lounging – Experimental animals were lounged for 30 min in trot with changes in their direction.
6. Racing – Horses took part in regional and international competitions. Average time that horses spent on the load continuously during the competition day was 60 min.

### 2.2 Blood collection and sampling

Experimental horses were subjected to blood sample collection at the beginning of the experiment, in the middle of the experiment and at the end of the experiment that lasted 1 year from *vena jugularis* using Hemos H – 02 tubes. Blood serum was centrifuged at 3000 rpm for 20 min and subsequently stored at −20 °C until the realisation of biochemical analysis [9]. Part of the uncoagulated blood was mixed in Eppendorf tubes with heparin for haematological analysis.

### 2.3 Analysis of haematological parameters

Hematologic parameters were analysed using automatic hematologic analyser – Abacus Vet5 (Diatron MI LDT, Budapest, Hungary) and following parameters were observed: WBC – Total count of leukocytes; LYM – Total count of lymphocytes; MID – Mid-size population of monocytes, basophils, eosinophils, blasts, and other immature cells; GRA – Total count of granulocytes; LYM % – Percent of lymphocytes; MID % – Percent of mid-size population of monocytes, basophils, eosinophils, blasts, and other immature cells; GRA % – Percent of granulocytes; RBC – Total count of erythrocytes; HGB – Haemoglobin; HCT – Haematocrit; MCV – Average volume of erythrocytes; MCH – Mean corpuscular haemoglobin; MCHC – Mean corpuscular haemoglobin concentration; RDWc – Red cell distribution width; PLT – Total count of platelets; PCT – Percent of platelets; MPV – Average volume of platelets; PDWc – Platelet distribution width [2].

### 2.4 Analysis of biochemical parameters

Biochemical analyses of blood serum were realised using commercial DiaSys kits and biochemical spectrophotometer RxMonza (Randox Laboratories Ltd., UK). Sodium, potassium and chlorides were measured using EasyLytePlus automatic analyser (The Hague, Netherlands). Mineral profile, energy profile, nitrogen profile and hepatic profile was analysed according to previous studies [2, 8, 9, 10, 11].

### 2.5 Statistical analysis

GraphPad Prism 6.1 programme was used for statistical analysis (6.1 version for Windows, GraphPad Software, La Jolla California USA (www.graphpad.com). Values were compared using ordinary one-way ANOVA method and the level of significance was set to: p < 0,5; p < 0,01; p < 0,001; p < 0,0001 and column statistics were calculated.

## 3 Results

### 3.1 Biochemical parameters

Statistically significant differences in the values of the mineral profile were found (Table 1). Calcium concentrations decreased significantly in the middle of the experiment compared to values measured before the experiment. In contrast, the values increased significantly at the end of the experiment, compared to values measured before and in the middle of experiment. Sodium values were significantly decreased after the experiment in comparison to the values measured at the beginning of the experiment. Exactly opposite results were spotted in concentrations of potassium, where a significant increase in concentrations at the end of the experiment, compared to the ones at the start was recorded.

**Table 1:** Parameters of mineral profile in blood serum of tested horses.

Parameter	Before the experiment	In the middle of experiment	After experiment	p-value
Ca (mmol/l)	3.014 ± 0.218^A,B^	2.642 ± 0.114^A,C^	3.484 ± 0.240^B,C^	∗∗∗∗^A,B,C^
P (mmol/l)	0.836 ± 0.151	0.908 ± 0.133	0.970 ± 0.282	ns
Mg (mmol/l)	1.051 ± 0.247	0.934 ± 0.084	0.978 ± 0.146	ns
Na (mmol/l)	142.5 ± 2.6^A^	140.6 ± 1.5	138.8 ± 1.8^A^	∗∗∗^A^
K (mmol/l)	3.55 ± 0.48^A^	3.77 ± 0.56	4.19 ± 0.41^A^	∗∗^A^
Cl^−^ (mmol/l)	102.9 ± 1.91	102.8 ± 1.43	101.7 ± 2.23	ns

Legend: ∗p < 0.05; ∗∗p < 0.01; ∗∗∗p < 0.001; ∗∗∗∗p < 0.0001.

∗Within the same row, means with same letters differ significantly.

In both monitored parameters of nitrogen profile, significant changes between individual samplings were observed (Table 2). Urea shown a demonstrable upward trend among successive groups. In the second monitored parameter (total proteins) a significant increase in concentration in the group at the end of the experiment compared to the group that was collected in the middle of the experiment was found.

**Table 2:** Nitrogen profile parameters in blood serum of tested horses.

Parameter	Before the experiment	In the middle of experiment	After experiment	p-value
Urea (mmol/l)	3.622 ± 0.373^A,B^	4.506 ± 0.824^A^	4.334 ± 0.736^B^	∗^A^;∗∗^B^
Total proteins (g/l)	62.97 ± 4.69	57.34 ± 9.38^A^	63.54 ± 3.21^A^	∗^A^

Legend: ∗p < 0.05; ∗∗p < 0.01; ∗∗∗p < 0.001; ∗∗∗∗p < 0.0001.

∗Within the same row, means with same letters differ significantly.

In parameters of energy profile, no statistically significant changes were observed (Table 3).

**Table 3:** Energy profile parameters in blood serum of tested horses.

Parameter	Before the experiment	In the middle of experiment	After experiment	p-value
Glucose (mmol/l)	5.922 ± 0.362	5.814 ± 0.261	5.508 ± 0.942	ns
TAG (mmol/l)	0.502 ± 0.116	0.427 ± 0.132	0.430 ± 0.138	ns

P > 0.05.

The parameters of hepatic profile (AST, ALT, GGT) had a statistically significant trend, in which the values in individual groups increased according to the elapsed time (Table 4).

**Table 4:** Hepatic profile parameters in blood serum of tested horses.

Parameter	Before the experiment	In the middle of experiment	After experiment	p-value
AST (μkat/l)	4.572 ± 0.369	4.247 ± 0.418^A^	5.035 ± 0.684^A^	∗∗∗^A^
ALT (μkat/l)	0.136 ± 0.043^A,B^	0.215 ± 0.068^A^	0.273 ± 0.075^B^	∗∗^A^;∗∗∗∗^B^
GGT (μkat/l)	0.242 ± 0.083^A^	0.410 ± 0.121^A^	0.313 ± 0.148	∗∗^A^
ALP (μkat/l)	3.673 ± 1.049	4.075 ± 0.856	4.329 ± 1.097	ns
Cholesterol (mmol/l)	2.043 ± 0.360	2.129 ± 0.357	2.186 ± 0.205	ns
Bilirubin (mmol/l)	23.83 ± 4.40	24.99 ± 7.80	23.72 ± 3.82	ns
Creatine kinase (μkat/l)	4.436 ± 1.950	4.995 ± 1.745	4.601 ± 2.504	ns

Legend: ∗p < 0.05; ∗∗p < 0.01; ∗∗∗p < 0.001; ∗∗∗∗p < 0.0001.

∗Within the same row, means with same letters differ significantly.

### 3.2 Hematologic parameters

During the experiment also hematologic parameters in experimental horses were observed – total count of leukocytes, granulocytes, lymphocytes, hematocrit, total count of thrombocytes etc. All measured values were within reference ranges and there were no visible health changes in horses throughout entire duration of experiment (Table 5).

**Table 5:** Hematological parameters in blood of tested horses.

Parameter	Before the experiment	In the middle of experiment	After experiment	p-value
WBC (10^9^/l)	5.997 ± 1.488^A^	5.962 ± 1.734^B^	8.002 ± 2.781^A.B^	∗^A,B^
LYM (10^9/^l)	2.287 ± 0.854	1.969 ± 0.938	2.079 ± 0.389	ns
MID (10^9^/l)	0.179 ± 0.188	0.320 ± 0.224	0.220 ± 0.201	ns
GRA (10^9^/l)	3.530 ± 1.153^A^	3.671 ± 0.903^B^	5.701 ± 2.538^A.B^	∗∗^A,B^
LY% (%)	38.62 ± 11.31^A^	32.31 ± 7.996	29.04 ± 10.95^A^	∗^A^
MI% (%)	2.938 ± 2.914	5.136 ± 3.079^A^	2.514 ± 2.211^A^	∗^A^
GR% (%)	58.44 ± 11.15^A^	62.54 ± 9.11	68.44 ± 10.27^A^	∗^A^
RBC (10^12^/l)	7.228 ± 0.716	6.829 ± 0.618	7.488 ± 0.867	ns
HGB (g/l)	144.10 ± 11.56	135.10 ± 8.20	145.60 ± 16.18	ns
HCT (%)	26.61 ± 2.05	25.18 ± 1.45^A^	27.55 ± 2.56^A^	∗^A^
MCV (fl)	36.85 ± 1.82	37.07 ± 2.06	37.00 ± 2.04	ns
MCH (pg)	19.98 ± 1.19	19.87 ± 1.11	19.48 ± 1.02	ns
MCHC (g/l)	541.1 ± 14.9	536.7 ± 9.9	528.0 ± 18.4	ns
RDWc (%)	24.73 ± 0.62	24.68 ± 0.71	24.58 ± 0.76	ns
PLT (10^9^/l)	64.85 ± 23.83^B^	77.07 ± 30.56^A^	106.80 ± 33.68^A.B^	∗^A^;∗∗^B^
PCT (%)	0.043 ± 0.016^B^	0.051 ± 0.020^A^	0.071 ± 0.023^A.B^	∗^A^;∗∗^B^
MPV (fl)	6.477 ± 0.667	6.714 ± 0.306	6.736 ± 0.506	ns
PDWc (%)	33.43 ± 3.41	33.83 ± 2.12	35.34 ± 2.04	ns

Legend: ∗p < 0.05; ∗∗p < 0.01; ∗∗∗p < 0.001; ∗∗∗∗p < 0.0001.

∗Within the same row, means with same letters differ significantly.

## 4 Discussion and conclusions

van der Kolk et al. [12] observed concentrations of calcium in horses with colic or diarrhoea in comparison to normal horses. Concentrations of calcium decreased from 3.1 mmol/l in normal horses to 2.9 mmol/l and 2.8 mmol/l respectively in horses suffering from colic or diarrhoea. In our study we observed concentrations at the level of 3.0 mmol/l to 3.5 mmol/l with a significant decrease in concentration in the middle of the experiment. Lower concentrations of Ca might have been caused by lower intake of feed during the summer period.

Effects of dietary energy and phosphorus content on blood chemistry and development of growing horses was observed in another study, where it was found that the concentrations of serum phosphorus varied from 1.86 mmol/l to 1.99 mmol/l [13]. In our study, the serum concentrations were about 50% from these values which can be caused by higher intake of phosphorus in energy based diets fed to growing horses as these are important in the younger period to ensure proper development.

Winter et al. [14] studied serum magnesium concentrations in horses and found that the mean concentration reached 0.71–0.81 mmol/l (within reference range for horses). Same as in our study, though our results were approximately 15% higher than in their study they state that there have been no abnormalities in the horses during clinical examination.

Another study focused on effects of dosages of NaCl on mineral status of exercising horses observed serum concentrations of chlorides in horses subjected to similar load as in our experiment [15]. Their findings shown concentrations ranged from 101 to 105 mmol/l which is in agreement with our results that ranged between 101.7 to 102.9 mmol/l over the course of the experiment and the values were within reference range. Similar results were found in our previous experiment (Massányi et al., 2020) where treadmill trained horses serum concentrations of chlorides were in the range of 101.8 mmol/l to 103.1 mmol/l.

Rueda-Carrillo et al. [16] measured concentrations of potassium in warmblood horses, their experimental group consisted of 28 animals and the results shown concentrations of 3.826 ± 3.168 mmol/l. Likewise, as their findings, our measured concentrations were in the same range of 3.55 ± 0.476 mmol/l and 4.19 ± 0.406 mmol/l. Another experiment conducted by our author collective observed concentrations of sodium in horses subjected to load on motion regulator HorseGym 2000 [10] and the measured concentrations in blood serum in individual experimental groups reached 139.2–142 mmol/l. In this experiment, where treadmill training was also one of the loads that animals have been subjected to shown concentrations ranging between 138.8 mmol/l to 142.5 mmol/l.

Brazilian sport horses under training in tropical climate in study executed by Padilha et al. [17] observed concentrations of urea and creatine kinase which were measured at levels of 4.91 mmol/l (urea) and 3.02 μkat/l (CK), whereas our results are in contradiction with those published by above-mentioned study with concentrations at levels of 3.622–4.506 mmol/l (urea) and 4.436–4.995 μkat/l (CK). There has been hardly any information published about the effects of climate on training of the horses but according to these results, it may be possible that it might have been affected by much more humid and warmer climate.

Total protein concentrations in our experiment ranged from 57.34 g/l in the middle of experiment to 63.54 g/l at the end of the experiment. Another study focused on training programmes by Arfuso et al. [18] shown concentrations to range between 59 to 62 g/l which is in consent with our findings, especially as the animals in both experiments were in the moderate levels of training program.

Brunner et al. [19] observed blood parameters in show jumping horses competing in Switzerland and found out that concentrations of glucose decreased to 3.6 mmol/l from original 4.3 mmol/l after the exercise. Opposing trend was found in concentrations of triglycerides, where concentrations rose from 0.26 mmol/l to 0.31 mmol/l after the exercise. In our study we have not compared pre- and post-training samples as collecting blood twice in such short time would cause increased stress to the animals but we found out that the concentrations of glucose over the course of our experiment ranged from 5.508 mmol/l to 5.922 mmol/l and triglycerides concentrations from 0.427 to 0.502 mmol/l. These changes can be caused by different diets or possibly more intensive training programmes which require higher overall glucose intake. All our measured values were within the reference range for warmblood horses.

Activity of hepatic parameters is a health state monitoring factor in all organisms. Increased activity of AST is usually a result of muscle or liver diseases [20]. In the cases, GGT activity is usually increased only mildly [21]. We have observed significant increase of AST in the sample collection after the experiment but there have not been any visible or significant changes to the horses' health state over the entire duration of the experiment and therefore we can state that it could've been affected by water ratio.

Study by Lassen and Swardson [22] observed hematological parameters in horses and set up reference ranges for blood. Our measured values were within reference ranges set by this study, except hemoglobin, which was approximately 15% lower. Another study on the same subject by Ono et al. [23] though shown that the concentrations of hemoglobin in Noma horse was 10.2–15.4 g/dl which agrees with our measured values. All other parameters that reference ranges were set for in this study also consent with our results. Therefore, we can state there can be certain fluctuations between various breeds of horses.

Based on presented results, we state that the one-year lasting experiment, during which animals were exposed to variety of experimental loads caused certain changes in internal environment of the animals. Though, there were no visible adverse effects to the individual animals, which consents that our experiment was executed properly and that the selected loads were adjusted properly for the animals, it is important to point out the importance of health state observation in experimental animals, as multiple internal and external factors can possibly affect the outcome of experiment and further study of this topic is highly encouraged.

## Declarations

### Author contribution statement

Martin Massányi: Conceived and designed the experiments; Performed the experiments; Analyzed and interpreted the data; Wrote the paper.

Marko Halo Jr.: Conceived and designed the experiments; Performed the experiments; Analyzed and interpreted the data.

Peter Massányi: Conceived and designed the experiments; Analyzed and interpreted the data; Contributed reagents, materials, analysis tools or data; Wrote the paper.

Eva Mlyneková: Performed the experiments; Analyzed and interpreted the data.

Agnieszka Greń; Grzegorz Formicki: Conceived and designed the experiments; Contributed reagents, materials, analysis tools or data.

Marko Halo: Analyzed and interpreted the data; Contributed reagents, materials, analysis tools or data; Wrote the paper.

### Funding statement

Peter Massányi was supported by Vedecká Grantová Agentúra MŠVVaŠ SR a SAV [VEGA 1/0698/22], Agentúra na Podporu Výskumu a Vývoja [APVV-21-0168].

Marko Halo was supported by Vedecká Grantová Agentúra MŠVVaŠ SR a SAV [VEGA 1/0392/20], Operational Programme Integrated Infrastructure within the project: Sustainable smart farming systems taking into account the future challenges [313011W112].

### Data availability statement

Data included in article/supp. material/referenced in article.

### Declaration of interest's statement

The authors declare no conflict of interest.

### Additional information

No additional information is available for this paper.
